# A Compensatory Role of NF-κB to p53 in Response to 5-FU–Based Chemotherapy for Gastric Cancer Cell Lines

**DOI:** 10.1371/journal.pone.0090155

**Published:** 2014-02-27

**Authors:** Fumitaka Endo, Satoshi S. Nishizuka, Kohei Kume, Kazushige Ishida, Hirokatsu Katagiri, Kaoru Ishida, Kei Sato, Takeshi Iwaya, Keisuke Koeda, Go Wakabayashi

**Affiliations:** 1 Molecular Therapeutics Laboratory, Iwate Medical University School of Medicine, Morioka, Japan; 2 Department of Surgery, Iwate Medical University School of Medicine, Morioka, Japan; 3 MIAST (Medical Innovation by Advanced Science and Technology) project, Iwate Medical University, Morioka, Japan; 4 Institute for Biomedical Sciences, Iwate Medical University, Yahaba, Japan; German Cancer Research Center, Germany

## Abstract

Despite of remarkable improvement of postoperative 5-FU–based adjuvant chemotherapy, the relapse rate of gastric cancer patients who undergo curative resection followed by the adjuvant chemotherapy remains substantial. Therefore, it is important to identify prediction markers for the chemotherapeutic efficacy of 5-FU. We recently identified NF-κB as a candidate relapse prediction biomarker in gastric cancer. To evaluate the biological significance of NF-κB in the context of 5-FU–based chemotherapy, we analyzed the NF-κB-dependent biological response upon 5-FU treatment in gastric cancer cell lines. Seven genes induced by 5-FU treatment in an NF-κB-dependent manner were identified, five of which are known p53 targets. Knockdown of *RELA*, which encodes the p65 subunit of NF-κB, decreased both p53 and p53 target protein levels. In contrast, NF-κB was not affected by *TP53* knockdown. We also demonstrated that cell lines bearing Pro/Pro homozygosity in codon72 of p53 exon4, which is important for NF-κB binding to p53, are more resistant to 5-FU than those with Arg/Arg homozygosity. We conclude that NF-κB plays an important role in the response to 5-FU treatment in gastric cancer cell lines, with a possible compensatory function of p53. These results suggest that NF-κB is a potential 5-FU-chemosensitivity prediction marker that may reflect 5-FU-induced stress-response pathways, including p53.

## Introduction

The majority of gastric cancer in the world is diagnosed in East Asia [1], where the standard therapy for advanced gastric cancers remains surgery and chemotherapy. Recently developed adjuvant chemotherapeutic regimens after curative gastrectomy for advanced gastric cancer have made remarkable progress in terms of controlling relapse and disease-free survival, particularly in the Japanese population [2], [3]. However, 30–40% of patients still experience relapse despite receiving chemotherapy after curative gastrectomy [3], suggesting that patient selection based on molecular information could potentially be very effective for increasing chemotherapy-mediated non-relapse and survival rates.

To select for gastric cancer patients who might benefit from chemotherapy, it is important to understand individual sensitivities before chemotherapy [4]. Post-operative adjuvant chemotherapy of gastric cancer provides an opportunity to test patient-derived tumors before they receive chemotherapy. In an attempt to identify potential biomarkers in this setting at the protein level, we previously reported a cell line panel screening system using quantitative protein expression profiling with Reverse-Phase Protein Arrays (RPPAs) [5], [6] combined with a cell-based growth assay system based on the concept of NCI-60 cell line screening panel [7], [8]. Candidate biomarkers were isolated based on correlation coefficients from protein expression and drug sensitivity matrix and then further validated using surgically-removed specimens [9]. Based on this approach we identified two biomarkers at the protein level, including NF-κB and JNK, whose levels had good correlation with chemotherapeutic response. The higher expression of NF-κB seemed to correlate with a poorer prognosis, while JNK showed an inverse correlation. These markers were also validated at the molecular level using gastrointestinal cancer cell lines. It has been shown that siRNA-mediated knockdown of p65 almost exclusively affects 5-FU sensitivity among currently-used chemotherapeutic drugs; but this is not the case for JNK knockdown [9]. Therefore, we concluded that NF-κB plays a dominant role in 5-FU treatment and JNK may be an indicator of chronic inflammation of the gastric background mucosae [10]. As an extension of this validation study, we sought to explore these proteins functionally and clarify the role of NF-κB as a stress-inducible transcription factor during 5-FU treatment. We also evaluated the role of p53 after 5-FU-mediated transactivation of NF-κB [10], [11] because it is well known that p53 is activated in response to this genotoxic agent [12]. In this study we report a potential compensatory role of NF-κB for p53 through analysis of a p53-NF-κB binding polymorphic site, codon 72 of p53. Together, these findings suggest that NF-κB/p53-codon72 could be a robust biomarker for 5-FU sensitivity.

## Materials and Methods

### Cell Lines

Nine human gastric cancer cell lines, including Kato-III, KE39, MKN74, MKN7, NUGC4, GSS, GCIY, and MKN45 were obtained from the RIKEN BioResource Center Cell Bank. IWT-1 was a *de novo* cell line that established in our laboratory from a Japanese male gastric cancer patient who had relapsed peritonitis carcinomatosa. The use of IWT-1 cell line has been approved by the Iwate Medical University Institutional Review Board (H25-116, and HG H25-15) and the family of donor patient who had died at the time of establishment of the cell line with a written informed consent with respect to taking the samples and making the cell line. Cells were grown to 70–80% confluency in RPMI-1640 supplemented with 10% fetal bovine serum (FBS) at 37°C in the presence of 5% CO_2._

### Preparation of Cell Lysate

Cells were harvested by centrifugation and cell pellets were lysed using Pink Buffer containing 9 M urea (Sigma-Aldrich, St. Louis, MI, USA), 4% 3-[(3-cholamidopropyl)dimethylammonio]-1-propanesul-fonate(CHAPS;Calbiochem, Merck Millipore, Darmstadt, Germany), 2% pH 8.0–10.5 pharma-lyte (GE Healthcare Japan, Tokyo, Japan), and 65 mM DTT (GE Healthcare Japan, Tokyo, Japan) as previously described [5], [13].

### Western Blot

SDS-PAGE was performed using NuPAGE 4–12% Bis-TrisGel electrophoresis (Invitrogen, Carlsbad, CA, USA), XCell Sure Lock Mini-cell (Invitrogen, Carlsbad, CA, USA), and Power PAC HC (BIO-RAD, Hercules, CA, USA). The resolved proteins on the gel were transferred to a nitrocellulose membrane using iBlot Dry Blotting System (Invitrogen, Carlsbad, CA, USA). The resulting membranes were blocked with 5% iBlot (Applied Biosystems, Foster City, CA, USA) and 0.1% Tween-20 (Bio-Rad, Hercules, CA, USA) in TBS (TBST) for 1 h. Membranes were then incubated with the indicated primary antibodies, including pan-actin, p53 (Thermo Scientific, Kalamazoo, MI, USA); p21, TIGAR, and PUMAα/β (Santa Cruz Biotechnology, Dallas, TX, USA); and NF-κB, α-tubulin, and PCNA (Cell Signaling Technology Japan, Tokyo, Japan). Next, the membranes were washed twice for 5 min with TBST, incubated with an HRP-conjugated secondary antibody for 1 h, and then washed twice for 5 min in TBST. Chemiluminescence detection reagents were incubated with the membranes for 1–5 min and then images were acquired using Image Quant LAS500 (GE Healthcare Japan, Tokyo, Japan). To evaluate protein induction by 5-FU, western blots were quantified using ImageJ (http://rsbweb.nih.gov/ij/).

### Immunocytochemistry

Cells were grown to 70–80% confluency in RPMI-1640 supplemented with 10% FBS in 4-chamber polystyrene vessel tissue culture-treated glass slides and then treated with 5-FU as indicated in each experiment. After the cells were exposed to 50 µM of 5-FU for 4 h to see an early transcriptional response, they were fixed in 4% paraformaldehyde, permeabilized using 0.2% Triton X-100 in PBS, and stained with DAPI (0.6 µM DAPI, 50 µl RNase, and 5 ml PBS) at room temperature for 12 min. Cells were then incubated with the following primary antibodies: anti-NF-κB p65, phospho-NF-κB p65 (Ser536), and phospho-p53 (Ser15) (Cell Signaling Technology Japan, Tokyo, Japan), and p53 (Thermo Scientific, Kalamazoo, MI, USA). Finally, the cells were incubated with either Alexa Fluor488- or 568-conjugated secondary antibody (Life Technologies Japan, Tokyo, Japan). A BX43 fluorescent microscope (Olympus, Tokyo, Japan) was used for image acquisition.

### Gene Expression Profiling

MKN45 cells were harvested after treatment with or without 50 µM of 5-FU for 4 h. RNA was then extracted from the harvested cells and gene expression profiling was performed according to the manufacturer’s instructions (Sure Print G3 Human GE8×60 K, Agilent Technologies Japan, Tokyo, Japan). Raw data were first normalized by dividing each probe signal by the 75^th^ percentile of the entire signal. Each microarray experiment was performed in duplicate resulting in two control and two 5-FU treatment microarray data sets. To identify genes that were differentially expressed in response to 5-FU, each control data set was compared separately to each 5-FU treatment set (4 comparisons). Differentially expressed genes were those that had a change in expression >2-fold in each comparison. We identified the final set of 10 differentially expressed genes based on their frequency in the 4 comparisons [14]. To confirm the reproducibility of these expression changes, quantitative real-time RT-PCR of 5-FU treated samples at 0, 4, 8, 12, and 24 h was performed for each gene. Primer sequences are listed in Table S1. For genes induced by 5-FU, analysis of promoter binding sites was performed using JASPAR algorithm (JASPAR, http://jaspar.genereg.net/). A 1000 bp promotor sequence specific to respective genes was obtained from Transcriptional Regulatory Element Database (http://rulai.cshl.edu/cgi-bin/TRED/tred.cgi?process=home). Binding sites were predicted by scanning promoter sequences with the consensus sequences of NF-κB and p53 with 70% of profile score threshold.

### RELA and TP53 Gene Knockdown

Cells were grown to 70–80% confluency in RPMI-1640 supplemented with 10% FBS in 6-well cell culture plates and then treated with NF-κB p65 or p53 siRNA (Cell Signaling Technology Japan, Tokyo, Japan) for 48 h. Briefly, knockdown was performed using Trans IT-TKO (Mirus Bio Corporation, Madison, WI, USA) at a concentration of 3% for 10 min at room temperature. Appropriate concentrations of siRNAs for each cell line was mixed with the Trans IT-TKO solutions followed by a 20 min incubation at room temperature. The siRNA concentrations used were as follows: 100 nM p65 siRNA for MKN45, and MKN74 cells, and 150 nM for GSS and Kato-III cells; and 100 nM p53 siRNA for MKN45, and GSS cells, and 50 nM for MKN74 cells. After 48 h, cells were harvested and protein levels were examined by Western blot. Two siRNA constructs possessing different sequences to the same target gene was used for each gene to confirm knockdown specificity. Cell cycle distribution was assessed using the Tali Image-based Cytometer (Life Technologies, Carlsbad, CA, USA). To see the maximum effect of siRNA on the 5-FU response, the drug was added 48 h after siRNA was transfected, and the respective cell cycle was measured after 24 h incubation with 5-FU. All experiments were repeated at least three times.

### TP53 Status and Codon72 Variant

DNA was extracted from gastric cancer cell lines using QIAmp DNA Mini Kit (Qiagen Japan, Tokyo, Japan). PCR amplification for the *p53* exon4 codon72 variant (Table S1) and the *p53* exon5–9 mutation was performed as previously described [15], [16]. Each PCR product was sequenced using the ABI PRISM 3030xl genetic analyzer (Applied Biosystems, Foster City, CA, USA) according to the manufacturer’s protocol. Sequencing results were analyzed using FinchTV (PerkinElmer Japan, Tokyo, Japan) and MEGA 5.1 Beta 3 [17].

### Growth Suppression Assay

Ten thousand cells per well were seeded in a 96-well microplate. Twenty-four hours later cells were treated with 5-FU for 24 h. After 5-FU treatment, the fraction of living cells was measured using the Cell Counting Kit-8 (Dojindo Molecular Technologies, Kumamoto, Japan) and a TriStar LB 941 microplate reader (Berthold Technologies, Bad Wildbad, Germany). Fifty percent growth inhibition concentration (GI_50_) was calculated using Prism software (Graph Pad Software, La Jolla, CA, USA). The GI_50_ values were used to determine correlations between 5-FU efficacy and protein levels based on the Pearson’s product-moment correlation coefficient (*r*).

## Results

### Fluorouracil Induces NF-κB

**Figure 1:**
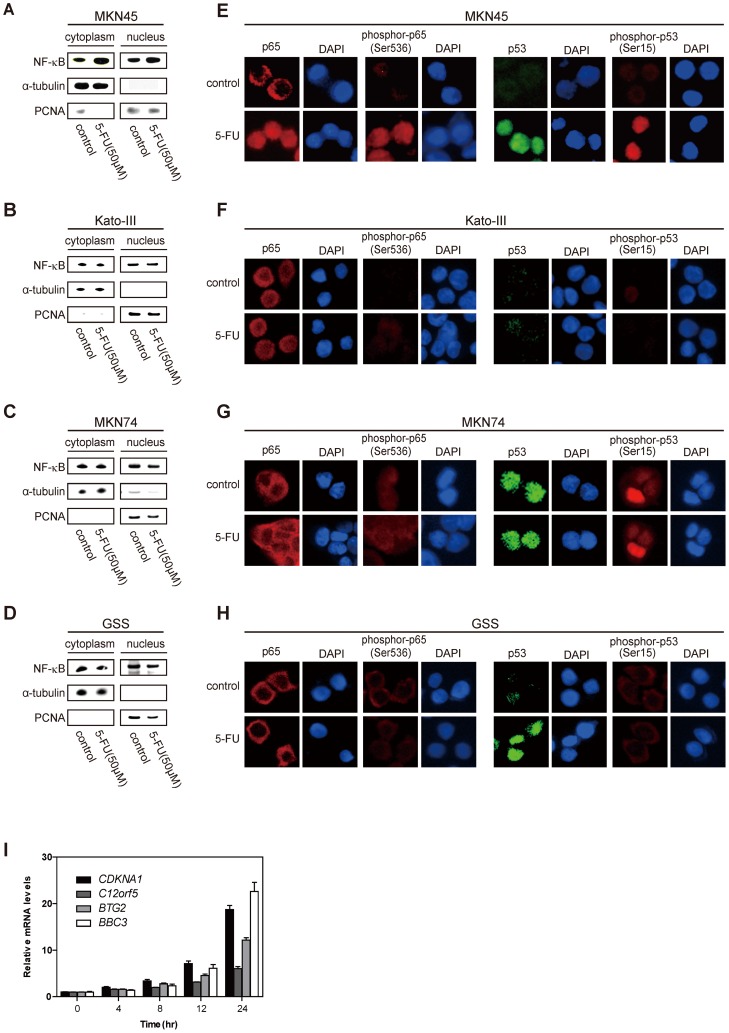
Induction and localization of NF-κB to the nucleus after 5-FU treatment in MKN45, Kato-III, MKN74, and GSS cells. A–D, Western blot analysis of the induction of NF-κB in the nucleus and the cytoplasm by 50 µM of 5-FU for 4 h for the indicated cell lines. PCNA and α-tubulin were used as the nuclear and cytoplasmic loading controls, respectively. E–H, Immunocytochemistry of p65 and p53 induction and localization by 5-FU treatment for the indicated cell lines. p65 (red), phosphor-p65 (Ser536; red), p53 (green), phospho-p53 (Ser15; red), and DAPI (blue) staining of the nucleus without (control) and with 5-FU treatment. I, Gene expression with quantitative real-time RT-PCR in a time course of 5-FU treatment. TIGAR, PUMA, CDKN1A, and BTG2 were identified as 5-FU induced genes. The quantitative values were relative to β-actin expression.

To confirm typical NF-κB behavior in response to 5-FU treatment, we traced the subcellular localization of NF-κB in the MKN45 (p53 wild type), MKN74 (p53 mutant), GSS (p53 mutant), and Kato-III (p53 homozygously deleted) cell lines. Western blot analysis with nuclear and cytoplasmic fractions demonstrated that 5-FU induced NF-κB in both compartments in MKN45 but was not observed in p53 mutant cell lines (Fig. 1A–D). We also examined the effect of 5-FU on NF-κB localization in the cells by immunocytochemistry (Fig. 1E–H). NF-κB localized to the cytoplasm in untreated MKN45, whereas 5-FU treatment caused an increase in NF-κB nuclear localization (Fig. 1E); however, no increase was observed in the p53 mutant cell lines (Fig. 1F–H). We also observed a drastic increase in phosphorylated NF-κB (p65 Ser536) in the nucleus of MKN45 treated with 5-FU, indicating NF-κB was transactivated by 5-FU (Fig. 1E). Constitutive nuclear localization and occasional phosphorylation of p53 was observed in MKN74 but did not seem to be induced by 5-FU (Fig. 1G). Nuclear localization of p53 was induced by 5-FU in GSS, but the activated signal was faint (Fig. 1H).

### p53 Targets are Induced upon 5-FU Treatment

Since NF-κB is a transcription factor (TF), its nuclear localization upon 5-FU treatment strongly suggests transactivation. We identified the top 7 transcripts among over 60,000 that were induced after 4 h of 5-FU treatment in MKN45 using gene expression profiling (Table 1). Interestingly, five of the 7 transcripts, namely *BBC3* (which encodes p53 up-regulated modulator of apoptosis, PUMA), *BTG2, C12orf5* (which encodes probable fructose-2,6-bisphosphatase TIGAR), *CDKN1A, and GPR87*, are known p53 targets [18]–[22]. The presence of promoter binding sites were predicted by scanning promoter sequences with the consensus sequences of NF-κB and p53 using JASPAR algorithm (Table 1) [23].

**Table 1:** Genes induced by 5-FU.

				Promoter binding site	
Genes (Alias)	p53 inducible(reference number)	Refseq	Promoter ID	NF-κB	p53
*BBC3(PUMA)*	Yes [20]	NM_014417.4	21819	Yes	Yes
*BTG2*	Yes [21]	NM_006763.2	1764	Yes	Yes
*C12orf5(TIGAR)*	Yes [19]	NM_020375.2	8600	Yes	No
*CDKN1A (p21/Cip1)*	Yes [18]	NM_000389.4	35406	Yes	Yes
*EDN2*	–	NM_001956.3	3557	Yes	No
*GPR87*	Yes [22]	NM_023915.3	30365	Yes	No
*GRIN2C*	–	NM_000835.3	18146	Yes	No

Promoter ID was obtained from http://rulai.cshl.edu/cgi-bin/TRED/tred.cgi?process=home.

We found that p53 levels were induced in the nucleus similar to those of NF-κB in response to 5-FU treatment (Fig. 1E). Treatment of 5-FU also increased the levels of p53 phosphorylation at Ser15, suggesting its transactivation (Fig. 1E, ref. [24]). In fact, the majority of total p53 induced by 5-FU seemed to be phosphorylated. We also observed a time-dependent induction of genes, including *C12orf5(TIGAR)*, *BBC3(PUMA)*, *CDKN1A(p21)*, and *BTG2* by 5-FU using RT-PCR (Fig. 1I). Taken together, these results suggest that the cellular response to 5-FU treatment may involve both NF-κB and p53 for transcriptional activation in this context, in MKN45.

### RELA Knockdown has a Greater Effect on p53 Target Proteins than TP53 Gene Knockdown

**Figure 2:**
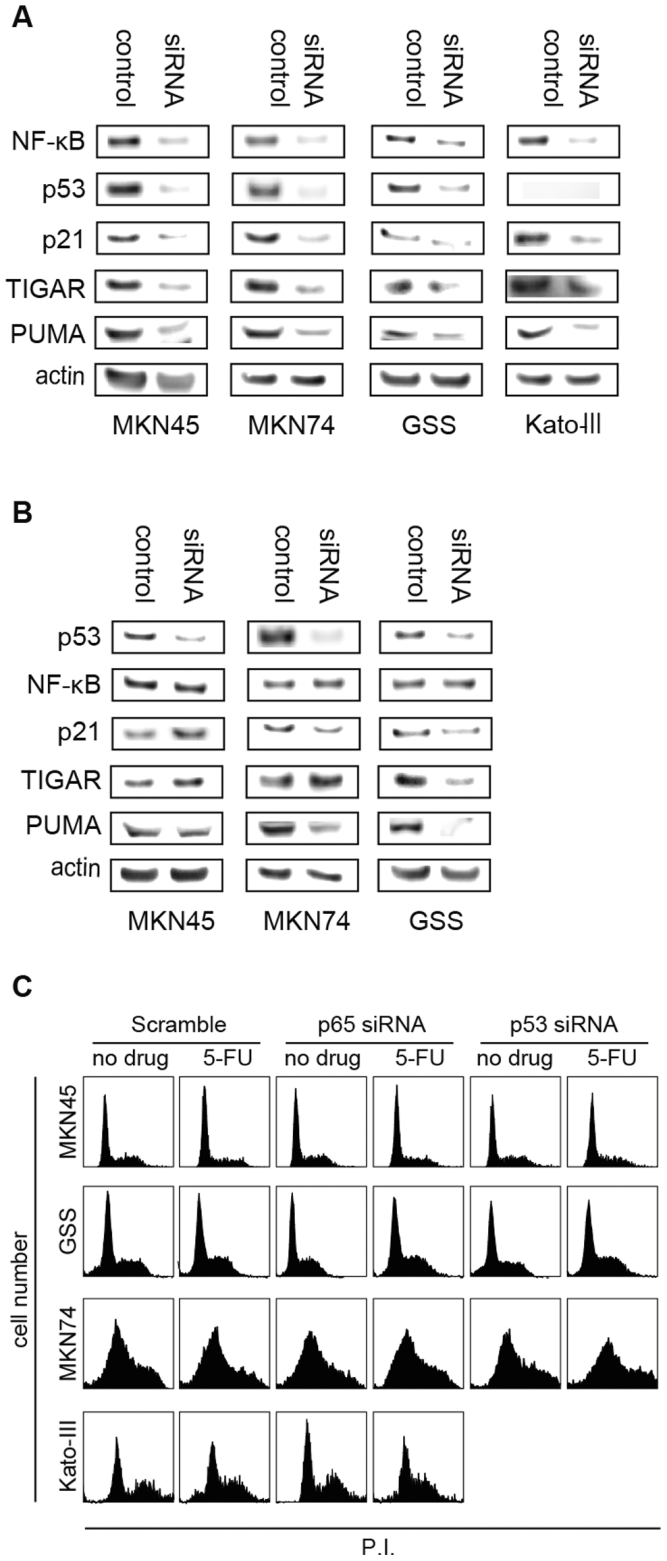
*RELA* and *TP53* knockdown. A, Western blot analysis of *RELA* knockdown in four gastric cancer cell lines. In addition to p53 and p65 proteins, p53 targets, including p21, TIGAR, and PUMA, were evaluated. Actin was used as a loading control. Results from cells incubated with RELA siRNA (siRNA) and without target siRNA (control) are shown. B, Western blot analysis of *TP53* knockdown in three gastric cancer cell lines. p53 allele of Kato-III is homozygously deleted so TP53 knockdown was not performed. C, Drug-induced cell cycle analysis. The horizontal axis, the strength of propidium iodine (P.I.), and the vertical axis indicate the cell numbers of each cell line.

To evaluate the regulatory effect of NF-κB and p53 in response to 5-FU treatment, we analyzed the protein levels of p65, p53, as well as known p53 targets, p21, TIGAR, and PUMA, by western blotting following *RELA* and *TP53* knockdown in MKN45, MKN74, GSS, and Kato-III. *RELA* knockdown caused a marked decrease in p53 levels in all cell lines. As expected, the levels of p21, TIGAR, and PUMA were also decreased (Fig. 2A). Conversely, while *TP53* knockdown decreased p53 levels, it did not affect p65 levels. As expected, the levels of p53 target proteins were decreased by *TP53* knockdown; however, this reduction was less than the reduction caused by *RELA* knockdown (Fig. 2B). In the cell cycle analysis, the MKN74, GSS, and Kato-III cell lines showed a slight increase in the S or G2 phase fraction whereas MKN45 exhibited an increase in the G1 fraction after 24 h exposure to 5-FU in the p65 and p53 knockdown similar to the corresponding scrambles (Fig. 2C). These results may indicate the robustness of the cellular stress response machinery that maintains the cell cycle distribution despite the knockdown of p65 and p53.

### TP53 Codon72 Pro Variant Exhibits Low 5-FU Sensitivity and High NF-κB Levels

Gene knockdown experimental results indicated that the interaction between NF-κB and p53 proteins might be important in the context of 5-FU treatment. To investigate the possibility that the *TP53* codon72 variant may affect cellular responses to 5-FU treatment, we sequenced *TP53* codon72 as well as mutations in the DNA binding domain coding regions (i.e., exons 5–8) of 9 gastric cancer cell lines (Table 2). The status of the codon72 variation and *TP53* mutation did not demonstrate clear associations.

**Table 2:** *TP53* status in gastric cancer cell lines.

	base change, position	Effect	codon72
Kato-III	NA	NA	NA
KE39	G to T (GTG to TTG)	Val to Leu	Arg/Arg
	exon8, codon272		
MKN74	A to C (ATC to CTC)	Ile to Leu	Arg/Arg
	exon7, codon251		
MKN7	wt	wt	Arg/Arg
NUGC4	wt	wt	Arg/Pro
IWT-1	C deletion (TCC to TCT)	Frameshift	Arg/Arg
	exon7, codon241		
GSS	T to G (CTT to CGT)	Leu to Arg	Pro/Pro
	exon6, codon194		
GCIY	T to G (CAT to CAG)	His to Gln	Pro/Pro
	exon5, codon179		
MKN45	wt	wt	Pro/Pro

NA, Not applicable; wt, wild type.

**Figure 3:**
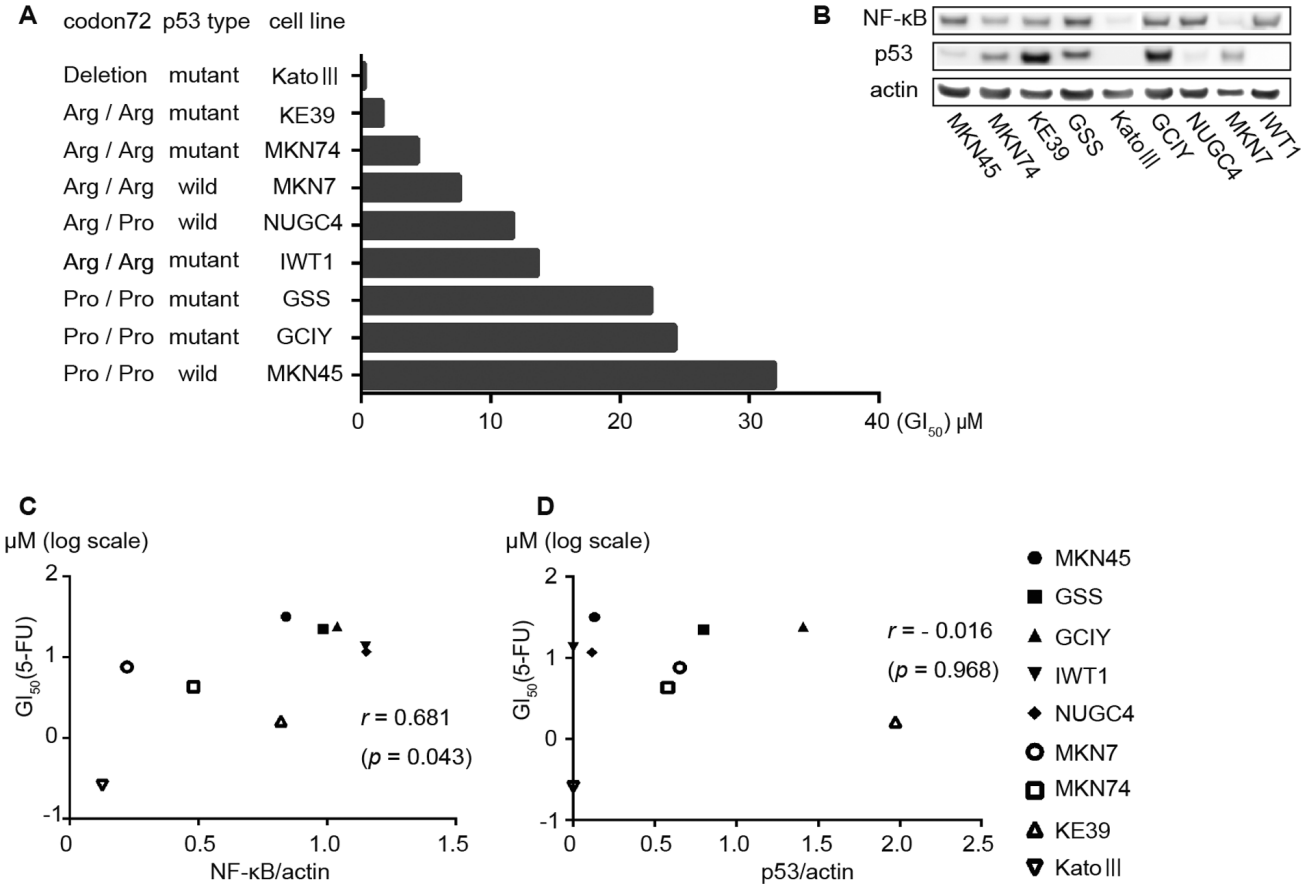
5-FU sensitivity of gastric cancer cell lines and their endogenous NF-κB and p53 levels. A, Growth suppression assay in 5-FU treatment in gastric cancer cell lines. Horizontal axes show GI_50_ value of 5-FU. B, Endogenous protein levels of NF-κB, p53, and actin by Western blot in gastric cancer cell lines. Actin is used as a loading control. C, Scattergram of the distribution of protein levels of NF-κB relative to actin and GI_50_ of 5-FU in each cell line. D, Scattergram of the distribution of p53 protein levels and GI_50_ of 5-FU in each cell line. Pearson’s product-moment correlation coefficient (*r*) as well as the *p* value is indicated in each scattergram.

We next investigated the association between 5-FU sensitivity and *TP53* status (Fig. 3A) as well as the endogenous levels of NF-κB and p53 (Fig. 3B). GSS, GCIY, and MKN45 lines, which possess the Pro homozygous variant, exhibited low sensitivity to 5-FU, whereas the KE39, MKN74, MKN7, NUGC4, and IWT1 lines possessing Arg allele exhibited relatively high sensitivity. Kato-III, which has a large *TP53* deletion [25], was the most sensitive to 5-FU. NF-κB protein levels were particularly correlated with 5-FU sensitivity (*r* = 0.68; *p* = 0.04; Fig. 3C); however, there was no clear correlation between p53 levels and 5-FU sensitivity (*r* = −0.04; *p* = 0.95; Fig. 3D). These results suggest that Pro homozygosity is associated with 5-FU resistance, while neither p53 mutation nor endogenous p53 levels directly affects the 5-FU sensitivity.

## Discussion

We have previously identified NF-κB as a potential prediction marker for post-operative 5-FU-based chemosensitivity for advanced gastric cancer [9]. NF-κB is an inducible transcription factor comprised of p65 (RelA), c-Rel, Rel-B, p50/NF-κB1, and p52/NF-κB2 [26], and plays a central role in immune responses and inflammatory cytokine regulation [27]–[29]. Chang et al previously demonstrated that NF-κB induced up or down regulation of differentially expressed genes in 5-FU-induced intestinal mucositis by inducing proinflammatory cytokines, such as IL-6, TNF-α, and IL-1β [10]. These 5-FU-induced inflammatory responses have been considered to be part of stress-avoiding processes that may lead to desensitization of 5-FU efficacy in gastric mucosa [30]. Although it has been suggested that activation of NF-κB is not directly associated with tumor development and progression [31], NF-κB has been considered to be a major biomarker and therapeutic target [32]. The direct evidence of reduced chemoresistance to 5-FU by siRNA for *RELA* together with the high discriminatory power of NF-κB nuclear staining in therapeutic outcomes of surgically removed tissues prompted us to perform further validation of NF-κB from a biological viewpoint.

Our transcriptional profiling results revealed that several p53 downstream genes were up-regulated in response to 5-FU, in which transactivation of NF-κB also occurred. These two major transcription factors have previously been shown to be co-regulated in response to genotoxic agents [33]–[35] and TNF-α [36], [37]. In addition, it has been proposed that co-activation of p53 and NF-κB in tumors treated with genotoxic agents could lead to therapeutic failure due to NF-κB-mediated survival signaling [38].

Individual knockdown of these transcription factors revealed genes downstream of p53 were affected by p65 knockdown, whereas the effect was limited by p53 knockdown. Previous reports have suggested a cooperative relationship between p53 and NF-κB in the context of autophagy, apoptosis, and S-phase checkpoint activation [34], [39]–[41]. Our results also indicated that NF-κB may compensate for the transcriptional activity of p53 when the intact function is lost in response to 5-FU treatment. In fact, a majority of gastric cancers bear mutations in the p53 DNA binding domain, thus rendering it transcriptionally inactive [42]. A recent study by Frank *et al*. reported that codon72 polymorphism of *TP53* substantially affects the ability of p53 to cooperate with NF-κB for gene transactivation, particularly in the induction of apoptosis through caspase 4/11 [40]. Together with the present finding that some transcriptional activity of p53 require NF-κB in response to 5-FU, p53 codon 72 polymorphism for its NF-κB binding may be more influential than mutational status of *TP53* or protein expression status of p53.

The impact of the codon72 polymorphism on spontaneous cancer risk has been previously investigated but not conclusively established due to limited human population and animal models [40], [43]–[45]. However, the codon72 polymorphism may play a role in maintaining established cancer cells than triggering cellular malignant transformation. In fact, previous studies have reported that the Pro/Pro allele is associated with resistance to chemotherapy and poor prognosis in the oral cavity [46] as well as colorectal [47], breast [48] and gastric [49] cancers and neuroblastomas [43]. A series of *in vitro* studies also support this hypothesis showing that the Arg allele is a more potent apoptosis inducer than the Pro allele [40], [50], [51]. Apoptosis is one of the major mechanisms induced by 5-FU and thus it is reasonable to hypothesize that the efficacy of 5-FU-based chemotherapy is associated with specific p53 polymorphisms [49]. Our *in vitro* findings support these epidemiological and experimental data and suggest a possible mechanism for the 5-FU-mediated p53-NF-κB interaction at the p53-codon72 binding site.

As expected, our study demonstrated that cell lines with the Pro allele were more resistant to 5-FU than those with the Arg allele. The growth suppression profile of 9 gastric cancer cell lines showed a good correlation to NF-κB protein levels. These results suggest that the Arg/Arg genotype has a stronger induction of apoptosis than the Pro/Pro genotype in the presence of 5-FU. Among the cell lines (all derived from Japanese gastric cancer patients), the ratio of Arg/Arg:Arg/Pro:Pro/Pro was 4∶1∶3, while that in healthy Japanese patients was 4.5∶4.4∶1 [52]. This may reflect a selection process that occurs during tumor development and the establishment as a cell line. Previous meta-analyses in cancer risk and p53-codon72 polymorphisms suggest that the Pro/Pro genotype has a higher cancer risk (lower for the Arg/Arg genotype) in Asian populations [45], [53]. However, the significance of “cancer risk” for cancer malignancy or treatment response remains to be elucidated, because it is generally difficult to conduct a clinical study dominated by genetic polymorphisms and assess the true genetic effects of treatment. To date, most reports describing an association between the p53 codon72 polymorphism and chemotherapeutic responses have demonstrated that the Arg/Arg genotype has a favorable response in a wide range of cancers treated with conventional genotoxic drugs [49], [54], [55]. We propose a putative mechanism for response to 5-FU via NF-κB and p53 protein binding associated with the p53 polymorphism, and thus a combinational diagnosis of NF-κB protein expression and codon72 may be a useful indicator for post-operative adjuvant chemotherapy. Aside from a few large scale datasets [52], [56], the extent of demographical and ethnic distributions of the polymorphism remains unclear. Accumulating data on ethnic differences for the polymorphism may explain the differences in cancer risk or chemotherapeutic response rate in patient population.

In summary, our findings indicate that NF-κB regulates p53 transcriptional activity in response to 5-FU, which may be associated with a polymorphic site of p53 at codon72. Further clinical and epidemiological studies should assess the utility of concomitant pathological/genetic evaluation of NF-κB/p53-codon72 in surgically-removed gastric cancer specimens in order to predict the efficacy of post-operative 5-FU-based adjuvant chemotherapy.

## Supporting Information

Table S1 Primer Sequences.
(DOCX)
