# Supplementary material for: A Compensatory Role of NF-κB to p53 in Response to 5-FU–Based Chemotherapy for Gastric Cancer Cell Lines
Source: PLoS One. 2014 Feb 27;9(2):e90155. doi: 10.1371/journal.pone.0090155 (PMC3937424; doi:10.1371/journal.pone.0090155)
Supplement: Table S1 — Primer Sequences. (DOCX) [file pone.0090155.s001.docx]

**Table S1 Primer sequences**

| Symbol (Alias) | Template | Primer | Sequence | Reference |
| --- | --- | --- | --- | --- |
| *CDKN1A (P21/Cip1)* | cDNA | Sense | GGCGGCAGACCAGCATGACAGATT | Jung, P. et al. PNAS, 105:15046-, 2008 |
|  |  | Antisense | GCAGGGGGCGGCCAGGGTAT |  |
| *C12orf5 (TIGAR)* |  | Sense | ATTCAGACAGCGGTATTCCAG | Original |
|  |  | Antisense | AAGCACCGTGACTCACAACTAA |  |
| *BTG2* |  | Sense | TGGGCTTAGGGAACCATCTCT | Original |
|  |  | Antisense | TTCAGCCAAGGAATACATGCAA |  |
| *BBC3 (PUMA)* |  | Sense | CCTGGAGGGTCCTGTACAATCT | Original |
|  |  | Antisense | GCACCTAATTGGGCTCCATCT |  |
| *ACTB (Actin, Beta)* |  | Sense | TCCCTGGAGAAGAGCTACGA | Original |
|  |  | Antisense | AGCACTGTGTTGGCGTACAG |  |
| *TP53 Condon72* | Genomic DNA | Sense | GTCCCCGGACGATATTGAAC | Original |
|  |  | Antisense | CGCCGGTGTAGGAGCTG |  |
| *TP53 Exon5* |  | Sense | TCTGTTCACTTGTGCCCTGAC | Kimura Y, et al. J Med Genet, 41:e57-, 2004 |
|  |  | Antisense | ATCAGTGAGGAATCAGAGGCC |  |
| *TP53 Exon6* |  | Sense | GCGCTGCTCAGATAGCGATG |  |
|  |  | Antisense | GGAGGGCCACTGACAACCA |  |
| *TP53 Exon7* |  | Sense | CTTGCCACAGGTCTCCCCAA | Kusser WC, PCR Methods and Appl, 2:250-, 1993 |
|  |  | Antisense | AGGGGTCAGCGGCAAGCAGA |  |
| *TP53 Exon8* |  | Sense | AAAGGACAAGGGTGGTTGGG |  |
|  |  | Antisense | CTGCACCCTTGGTCTCCTCC |  |
| *TP53 Exon9* |  | Sense | CGGTGGAGGAGACCAAGGGT |  |
|  |  | Antisense | AAGAAGAAAACGGCATTTTG |  |
